# Premorbid adjustment amongst outpatients with schizophrenia in a Nigerian psychiatric facility

**DOI:** 10.4102/sajpsychiatry.v27i0.1492

**Published:** 2021-05-28

**Authors:** Omokehinde O. Fakorede, Adegboyega Ogunwale, Akinwande O. Akinhanmi

**Affiliations:** 1 Department of Mental Health and Behavioural Medicine, Federal Medical Centre, Abeokuta, Nigeria; 2 Forensic Unit, Neuropsychiatric Hospital, Abeokuta, Nigeria; 3 Department of General Adult Psychiatry and Drug Addiction Treatment, Neuropsychiatric Hospital, Abeokuta, Nigeria

**Keywords:** premorbid adjustment, premorbid functioning, schizophrenia, psychosis, functioning, out-patients, Nigeria

## Abstract

**Background:**

Studies from developed countries have shown that poor premorbid adjustment in patients with schizophrenia is associated with poor outcome. However, similar studies in developing countries like Nigeria are few despite the stability of schizophrenia prevalence across cultures.

**Aim:**

The aim of this study was to assess the prevalence and correlates of poor premorbid adjustment amongst outpatients with schizophrenia.

**Setting:**

The Neuropsychiatric Hospital, Abeokuta in Ogun State, Nigeria.

**Methods:**

The premorbid adjustment of 300 outpatients with schizophrenia was assessed using the premorbid adjustment scale. Pattern and severity of psychosis, overall illness severity, global assessment of functioning and socio-demographic factors were investigated as correlates of premorbid functioning.

**Results:**

About half (53.3%) of the respondents had poor premorbid adjustment and most of them were males (56.9%). Poor premorbid adjustment was associated with male gender (*χ*^2^ = 7.81, *p* = 0.005) whilst good premorbid adjustment was associated with no or borderline illness severity (*χ*^2^ = 8.26, *p* = 0.016) as well as no or mild impairment in functioning (*χ*^2^ = 7.01, *p* = 0.029) amongst the respondents. Positive, negative and general symptomatology were predicted by premorbid adjustment at different developmental stages.

**Conclusion:**

Consistent with existing literature, poor premorbid adjustment was prevalent amongst patients with schizophrenia in this study and was associated with male gender, poorer clinical outcomes and greater illness severity. Mental health promotion and other preventative approaches are recommended as possible early intervention strategies in dealing with schizophrenia.

## Introduction

Premorbid adjustment in relation to mental disorder has been defined by various authors. Philips^1^ defined the concept as the extent to which an individual had fulfilled the appropriate expectations considering gender and age prior to the development of the illness, whilst Cannon-Spoor^2^ described it as a measure of an individual’s attainment of age-appropriate developmental and social milestones from childhood until about 6 months before onset of illness. Scholars have reiterated the prognostic importance of premorbid adjustment in schizophrenia.^2,3^

Generally, it has been shown that most patients with schizophrenia had poor premorbid adjustment,^4,5^ and this has been associated with brain atrophy,^6^ ventricular enlargement,^7^ attention deficits,^8^ verbal reasoning difficulties,^9^ negative symptoms,^10^ poor treatment response,^11^ prolonged hospital stay^12^ and poor functioning^13^. It is important to note that the above findings were observed amongst people of European descent and there appears to be only one relevant study on the subject amongst Nigerian patients with schizophrenia.^14^ It was a comparative study on the premorbid social adjustment of 38 patients with schizophrenia and 20 with mania. The authors found that the patients with schizophrenia consistently tended to have poorer premorbid adjustment compared with patients with mania, and this was significant with the domain of ‘highest level of functioning’. Amongst the group with schizophrenia, the men had poorer premorbid functioning than the women, and poor premorbid functioning was associated with the number of years of formal education.

Some of the limitations of this work are the rather small sample size and narrow investigation for possible correlates of poor premorbid adjustment. Whilst the study above did not assess scholastic and occupational premorbid functioning for some developmental stages, utilised a small sample size of 58 in all and compared the findings amongst two clinical groups – schizophrenia and mania – there is a need to carry out a study that only focuses on schizophrenia, is comprehensive (assesses beyond the social functioning of the patients) and explores the relationship between the variable of interest and significant clinical parameters (psychosis pattern illness severity and current functioning). These correlates may aid the retrospective diagnosis of poor premorbid adjustment as well as prognosticate illness outcome in patients with schizophrenia. Findings on pre-psychotic and early psychotic illness constitute significant prognostic parameters in the management of psychotic disorders. However, such studies amongst Nigerian patients with schizophrenia are few. Therefore, studies on schizophrenia and related concepts from Nigeria becomes imperative for scientific knowledge and comparative purpose. Our study therefore aimed to study ‘premorbid adjustment’ and its correlates amongst a sample of Nigerian patients with schizophrenia, as well as investigate the contribution of the clinical variables at different socio-sexual stages of premorbid functioning.

## Methods

This cross-sectional research was part of a larger study conducted amongst outpatients with schizophrenia, at the Neuropsychiatric Hospital, Abeokuta in Ogun State, Nigeria. Individuals who fulfilled the inclusion criteria were recruited (through consecutive sampling method) for the study over a 3-month period (January–March 2014). The inclusion criteria were being within the age bracket of 18–64 years, having a clinical diagnosis of schizophrenia and having been an outpatient of the hospital for at least 1 year.

An estimated sample size of 256 was derived based on the prevalence of 78% of social disability amongst outpatients with schizophrenia in the same study setting 21 years ago.^15^ This was calculated using the Cochran’s minimum sample size formula^16^:

$$n=\frac{Z^{2}pq}{d^{2}}$$ [Eqn 1]

where:

*n* = the desired sample size when the population is > 10 000 *Z* = the standard normal deviate, 1.96 at 95% confidence level *p* = the proportion of social disability amongst outpatients with schizophrenia, 78%^15^

*q* = 1.0 – *p* = 0.22

*d* = degree of accuracy desired usually set at 0.05.

Therefore:

$$n=\frac{1.96\times 1.96\times 0.78\times 0.22}{0.05\times 0.05}$$ [Eqn 2]

*n* = 263.68

However, because the study population is below 10 000, the true sample size (*n*_*f*_) was estimated as:

$$n_{f}=\frac{n}{1+\frac{\left(n\right)}{\left(N\right)}}$$ [Eqn 3]

where:

*n*_*f*_ = the desired sample size when the study population is less than 10 000

*n* = the desired sample size when the study population is more than 10 000, that is, 264

*N* = the estimated study population which is 9417, the population of outpatients within the age of 18–64 years and with the diagnosis of schizophrenia in the year 2010.^17^

Therefore:

$$n_{f}=\frac{264}{1+\frac{264}{9417}}$$ [Eqn 4]

***nf* = 256**

This was then oversampled by 15% to account for refusal and non-response, which gave a total of **294.4–300**.

During the data collection, a total number of 316 patients who met the inclusion criteria were approached. Using the World Health Organization International Classification of Diseases as a diagnostic instrument to confirm the diagnosis of schizophrenia amongst the respondents, 11 did not meet the diagnostic criteria whilst 5 did not give consent to participate. The remaining 300 patients who were positive for schizophrenia and gave their consent were recruited for the study.

The study was approved by the Neuropsychiatric Hospital Aro Health Research Ethics Committee, and all respondents consented to participate in the study. Patients who met the inclusion criteria were assessed for their socio-demographic factors, level of premorbid adjustment, severity and pattern of psychosis, overall severity of psychosis and their current level of functioning in that order.

Participant’s socio-demographic and clinical characteristics were assessed using the socio-demographic questionnaire which contained close-ended questions.

The premorbid adjustment scale (PAS)^18^ was used to assess the level of premorbid adjustment of the patients in this study. The scale has five subscales – childhood (up to age 11), early adolescence (12–15 years), late adolescence (17–18 years), adulthood (19 years and above) and general subscales. The general subscale is not particularly useful in research because it does not assess premorbid function per se, and it is biased towards the young age group.^19^ Therefore, it was also not used in this work. Previous authors used the Israeli Draft Board’s Assessment as a comparative tool with the PAS and the latter demonstrated good predictive and concurrent validity. It yielded 0.76 and 0.80 for the social-related scales and 0.71 and 0.72 for the academic-related scales.^12^ Furthermore, a Nigerian study by Gureje et al.^14^ reported a value of 0.773 as the Cronbach’s α for internal consistency. The adapted instrument used in this work contains 17 items each of which has a phrased description for scoring. Each scale assesses an individual’s social accessibility, peer relationships, functioning beyond the nuclear family and capacity to form intimate socio-sexual ties prior to the onset of the illness. The scale’s reliability using Cronbach’s α ranged between 0.72 and 0.79. Scores for each subscale is calculated by dividing the obtained score by the total obtainable score for that subscale. The overall score is an average of the scores obtained on all the developmental scales that apply to each individual. Each item on each subscale is scored on a Likert scale of 0 to 6 with 0 and 6 representing the ‘healthiest’ and ‘least healthy’ ends, respectively. The median PAS score in this sample was found to be 0.29. Scores below 0.29 were categorised as good premorbid adjustment whilst scores of 0.29 and above are grouped as poor premorbid adjustment.^20^ A Nigerian study which validated the instrument reported an (Cronbach’s) internal consistency of 0.773.^14^

The positive and negative syndrome scale (PANSS)^21^ was used to assess the pattern of psychosis (positive symptoms, negative symptoms or general psychiatric symptoms). The PANSS is a 30-item, 7-point rating instrument with 18 items adapted from the brief psychiatric rating scale^22^ and 12 from the psychopathology rating schedule.^23^ It relates the positive and negative symptoms of schizophrenia to the global psychopathology. Thirty items are subdivided into positive (7 items), negative (7 items) and general psychopathology (16 items) subscales, with each item rating point representing increasing levels of psychopathology: 1 = absent, 2 = minimal, 3 = mild, 4 = moderate, 5 = moderate severe, 6 = severe and 7 = extreme. The score ranges from 7 to 49 for both the positive and negative subscales and 16 to 112 for the general psychopathology subscale. The subtotal scores for each of the subscales were calculated and then summed to make the total score. The minimum total score is 30 and the maximum total score is 210. Higher scores reflect higher severity of psychosis. At its development, the *a* coefficients for the positive and negative scales were found to be 0.73 and 0.83, respectively.^24^ For the positive, negative, composite and general psychopathology scales, the test–retest Pearson correlations were 0.80 (*p* < 0.001), 0.68 (*p* < 0.01), 0.66 (*p* < 0.01) and 0.60 (*p* < 0.02), respectively.^25^ In a South African study, the inter-rater reliability was also found to be good, *r* = 0.88.^26^ The instrument has, however, been used amongst Nigerian patients with schizophrenia.^27^ The PANSS test–retest reliability obtained in our preliminary validation study for the positive, negative and total subscales was good (*n* = 0.985, 0.980 and 0.797, *p* < 0.001, respectively).

The severity subscale of the clinical global impression (CGI)^28^ measures the overall severity of psychosis. It provides information as regards the overall functioning of patients either prior to or after instituting medications. The rater is expected to consider the patient’s history, symptoms, illness severity, level of distress, behaviour and the impact of the symptoms on the patient’s functioning. The CGI has three subscales: the first (CGI-Severity, CGI-S) rates illness severity; the second, CGI-Improvement (CGI-I) rates the change from the initiation of treatment; and the third rates the efficacy index (CGI-E), which measures the therapeutic response. Only CGI-S is useful for a cross–sectional study^29^ as the current work. The other subscales are useful in prospective and drug-intervention studies, respectively.^29^ With the CGI-S, ratings are done on a 7-point scale of 1–7 (1 = normal, not at all ill; 2 = borderline mentally ill; 3 = mildly ill; 4 = moderately ill; 5 = markedly ill; 6 = severely ill and 7 = amongst the most extremely ill patients) based upon observed and reported symptoms, behaviour and function in the past 7 days. It has been shown to correlate well with other standard instruments such as the brief psychiatric rating scale^30^ and has good psychometric properties: high face validity^28^ and good inter-rater reliabilities of 0.66 and 0.51 for the CGI-S and the CGI-I subscales, respectively.^30^ It has been extensively used^31^ even in this environment.^32^ The test–retest reliability we conducted in our preliminary validation study was 0.860, *p* < 0.001.

The global assessment of functioning (GAF)^33^ was used to evaluate the symptom description and socio-occupational functioning in this population sample. It is a product of serial modifications of the health-sickness rating^34^ which was later revised to the global assessment scale.^35^ The GAF was eventually introduced, after the global assessment scale, as a new rating scale for the assessment of the overall psychiatric disturbance on Axis V of the Diagnostic and Statistical Manual of Mental Disorders^33^ (American Psychiatric Association, 1987). It is a 100-scale instrument which is divided into 10 ranges of functioning of anchor descriptions, with each decile having two components: general descriptions of symptom severity and behavioural descriptors of social–occupational functioning. The patient is rated relative to the deciles within which he or she falls, depending on the symptom severity or the level of function within that range. The exact GAF score is then determined from within the decile and the final score is the most severe assessment of either the psychological symptoms or the social–occupational level of function. A score of 0 is assigned if there is not enough information to make an assessment. In a study conducted by Hilsenroth et al.^36^ at a university-based outpatient community clinic, its inter-rater reliability was found to be excellent (*r* = 0.86) whilst its relationship to the global assessment of relational functioning was significant (*r* = 0.60, *P* < 0.0001) as was its relationship to the social and occupational functioning assessment scale (*r* = 0.60, *P* < 0.0001). This instrument has been used in various studies in this environment.^37,38^ In this work, the score obtained for each respondent was categorised into no to mild impairment (61–100), serious to moderate impairment (41–60) and severe impairment (1–40) in functioning. The test–retest reliability we conducted in a preliminary validation study was 0.943, *p* < 0.001.

## Statistical analysis

The Statistical Package for Social Sciences (version 16.0) was used to analyse the data obtained in this work.

Categorical data from qualitative variables were expressed as percentages of the total whilst continuous data of numerical variables were expressed in means (and standard deviations). However, data of ‘premorbid adjustment’ which are continuous were further dichotomised into ‘good’ and ‘bad’ adjustment using the median score.^39^

Chi-square analysis was done to determine the association between premorbid adjustment and the other variables (both socio-demographic and clinical).

The variables that demonstrated significance in the tests of association (gender, illness severity and global assessment of functioning) were entered as predictor variables in a multivariate logistic regression, whilst the outcome variable remained the premorbid adjustment. The derived logistic regression odds ratio was presented with 95% confidence intervals.

The contribution of premorbid adjustment at different socio-sexual stages to the clinical variables was investigated using multiple linear regression.

The level of significance for all statistical tests was *p* < 0.05.

## Results

The study respondents were 300 in total: 148 males and 152 females. The mean age of the respondents was 41.9 (standard deviation [SD] = 10.1) years, with a range of 20–64 years and most were of the Yoruba tribe (92.7%), Christians (74.4%), unmarried (69.3%) with secondary level of education (43.3%) and employed (Table 1).

**TABLE 1:** The socio-demographic characteristics of the respondents.

Variables	*N*	%
**Gender**		
Male	148	49.3
Female	152	50.7
**Age (years)**		
20–39	136	45.4
40–59	149	49.6
60 and above	15	5.0
Mean ± SD	41.9 ± 10.05	
**Ethnicity**		
Yoruba	278	92.7
Other tribes	22	7.3
**Religion**		
Christianity	223	74.4
Islam	76	25.3
Trado-African	1	0.3
**Marital status**		
Single	119	39.7
Married	92	30.7
Separated/divorced	66	22
Widowed	23	7.6
**Highest educational status**		
None	16	5.3
Primary	56	18.7
Secondary	130	43.3
Tertiary and postgraduate	98	32.7
**Employment status**		
Unemployed	100	33.3
Student/apprentice	24	8.0
Employed	168	56
Retired	8	2.7

The mean age (SD) of the onset of illness, duration of illness, duration of untreated psychosis, number of episodes, number of previous hospitalisation, total PANSS score and GAF score were 28.4 ± 8.5 (14–60) years, 13.5 ± 8.6 (2–40) years, 187.03 ± 155.05 (6–1300) weeks, 2.6 ± 1.0 (0–5), 0.9 ± 1.0, 33.85 ± 8.27 (30–106) and 76.25 ± 14.57 (21–85), respectively (Table 2).

**TABLE 2:** Clinical characteristics of the respondents.

Variables	Mean	SD
Age at illness onset (years)	28.4	8.5
Total illness duration (years)	13.5	8.6
DUP (weeks)	187	155.1
Number of episodes	2.6	1.0
Number of previous hospitalisation	0.9	1.0
**PANSS**		
Positive subscale	8.7	3.7
Negative subscale	8.5	3.8
General subscale	16.6	2.7
Total	33.9	8.3
CGI	1.76	1.2
GAF	76.3	14.6
**Overall premorbid adjustment**		
Mean	0.34	0.2
Median (IQR)	0.29	0.2
Childhood premorbid adjustment	0.32	0.2
Early adolescence premorbid adjustment	0.36	0.1
Late adolescence premorbid adjustment	0.34	0.1
Adulthood premorbid adjustment	0.28	0.2

DUP, duration of untreated psychosis; PANSS, positive and negative syndrome scale; CGI, clinical global impression; IQR, interquartile range; SD, standard deviation.

**FIGURE 1:**
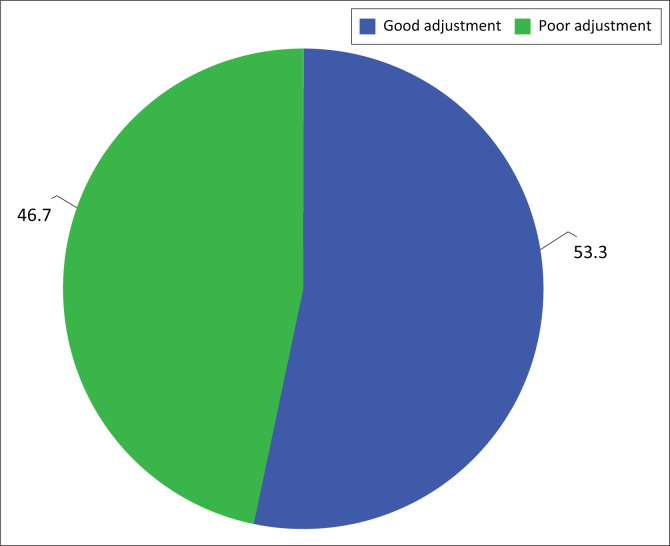
Overall premorbid adjustment.

The mean overall premorbid adjustment score was 0.34, whilst the median was 0.29. Using the median to split into ‘good’ and ‘poor premorbid’ adjustment, 53.3% of the respondents had poor premorbid adjustment (Figure 1).

Poor premorbid adjustment was significantly associated with the male gender, whilst good premorbid adjustment was associated with no to borderline illness as well as no to mild impairment (Table 3).

**TABLE 3:** Socio-demographic and clinical correlates of premorbid adjustment.

Variables	Good premorbid adjustment		Poor premorbid adjustment		*χ*^2^	*df*	*p*
	*n*	%	*n*	%			
**Gender**							
Male	57	40.7	91	56.9	7.81	1	0.005*
Female	83	59.3	69	43.1	-	-	-
**Highest educational achievement**							
Below tertiary education	94	67.1	108	67.5	-	-	-
Tertiary education and above	46	32.9	52	32.5	0.04	1	0.948
**Employment status**							
Employed	83	59.3	85	53.1	1.15	1	0.284
Unemployed	57	40.7	75	46.9	-	-	-
**Severity of illness**							
Not ill/borderline	118	84.2	113	70.6	8.26	1	0.016*
Mild/moderate	18	12.9	35	21.9	-	-	-
Marked/severe	4	2.9	12	7.5	-	-	-
**History of hospitalisation**							
Yes	75	53.6	73	45.6	1.89	1	0.17
No	65	46.4	87	54.4	-	-	-
**Global assessment of functioning**							
No/mild impairment	128	91.5	129	80.6	-	-	-
Moderate impairment	10	7.1	5	3.2	7.01	2	0.029*
Severe impairment	2	1.4	26	16.2	-	-	-

* , *p* < 0.05.

Of the three variables investigated, only the gender added significantly to the prediction (Table 4). The odds of having a poor premorbid adjustment was twofold greater for males as opposed to females (Table 4).

**TABLE 4:** Independent correlates of premorbid adjustment.

Variable	*B*	SE	Wald	Sig.	OR	95% CI		Nagelkerke *R*^2^
						Lower	Upper	
**Gender**								
Male	0.814	0.254	10.262	0.001	2.3	1.37	3.71	0.078*
Female	-	-	-	-	-	-	-	-
Severity of illness	0.162	0.233	0.486	0.486	1.2	0.75	1.86	-
Global assessment of functioning	−0.122	0.207	0.346	0.557	0.89	0.59	1.33	-

* , *p* < 0.05.

As shown in Table 5, multiple linear regression models were estimated to predict the severity of psychosis (based on PANSS scores), overall illness severity and current level of functioning from four premorbid developmental stages amongst the participants. With regard to the positive subscale of the PANSS, the developmental stages accounted for only 6.7% of the variability of the positive symptomatology. However, only the adulthood stage demonstrated a significant prediction to the model (*p* = 0.003). Negative PANSS score was independently predicted by childhood, late adolescence and adulthood premorbid functioning (*p* = 0.000), explaining 16.8% of the variability in the negative symptomatology. In terms of general PANSS, the developmental stages only accounted for 6.4% of the variability in the non-specific symptomatology.

**TABLE 5:** Linear regression modelling of the association between socio-sexual stages of premorbid adjustment and illness severity.

Variable	β†	*t*	p*	*F*	p*	*R*^2^
**Positive subscale PANSS**				4.775	0.001	0.067
Childhood	0.167	1.652	0.100			
Early adolescence	0.12	0.991	0.322			
Late adolescence	−0.335	−2.804	0.005*			
Adulthood	0.242	3.016	0.003*			
**Negative subscale PANSS**				13.442	0	0.168
Childhood	0.427	4.467	0.000*	-	-	-
Early adolescence	0.061	0.5333	0.595	-	-	-
Late adolescence	−0.401	−3.555	0.000*	-	-	-
Adulthood	0.275	3.634	0.000*	-	-	-
**General subscale PANSS**				5.639	0	0.078
Childhood	0.345	3.43	0.001*	-	-	-
Early adolescence	−0.047	−0.392	0.695	-	-	-
Late adolescence	−0.407	−3.431	0.001*	-	-	-
Adulthood	0.207	2.6	0.010*	-	-	-
**PANSS total**				10.861	0	0.14
Childhood	0.377	3.874	0.000*	-	-	-
Early adolescence	0.067	0.576	0.565	-	-	-
Late adolescence	−0.46	−4.016	0.000*	-	-	-
Adulthood	0.299	3.88	0.000*	-	-	-
**CGI**				3.557	0.08	0.051
Childhood	0.152	1.49	0.138	-	-	-
Early adolescence	0.028	0.232	0.817	-	-	-
Late adolescence	−0.167	−1.383	0.168	-	-	-
Adulthood	0.204	2.519	0.012*	-	-	-
**GAF**				4.246	0.002	0.06
Childhood	−0.193	−1.897	0.059	-	-	-
Early adolescence	−0.029	−0.24	0.811	-	-	-
Late adolescence	0.194	1.622	0.106	-	-	-
Adulthood	−0.207	−2.568	0.011*	-	-	-

* , *p* < 0.05.

† , Standardised β.

PANSS, positive and negative syndrome scale; CGI, clinical global impression; GAF, global assessment of functioning.

Childhood and late adolescence were statistically significant in terms of their predictive power (*p* = 0.001). Total PANSS scores were predicted by childhood, late adolescence and adulthood premorbid functioning (*p* = 0.001). The premorbid adjustment explained 14% of the variability in the global psychotic phenomena.

The prediction model for the relationship between premorbid functioning and global illness severity was not significant (*F* = 3.56, *p* = 0.08) although adulthood premorbid functioning significantly explained 5.1% of the variance in illness severity (*p* = 0.012). Similarly, global functioning was predicted by adulthood premorbid functioning scores (*p* = 0.011) and the overall model was significant (*F* = 4.25, *p* = 0.002). Premorbid functioning accounted for 6% of the variance in global functioning.

## Discussion

This study aimed to determine the prevalence of poor premorbid adjustment as well as its correlates amongst Nigerian patients with schizophrenia. It represents one of the few studies that have focused on the subject amongst patients in the developing countries.

The prevalence of poor premorbid adjustment in this sample is slightly more than 50% and is consistent with earlier studies conducted amongst Nigerians^14^ and non-Nigerians.^4,5,18^ A possible explanation as proposed in some studies is the role of the neurodevelopmental hypothesis.^40,41,42^ These authors posited that insults to the immature brain (during the premorbid period) result in non-progressive damage to the brain with resultant psychotic symptoms characteristic of schizophrenia. However, why a substantial number of patients had good premorbid adjustment and still had the disease may be accounted for by other aetiological factors in schizophrenia as explained by its multifactorial basis.^43,44^

Of all the variables investigated, poor premorbid adjustment was significantly associated with male gender. Studies^14,45,46,47^ have shown that males with schizophrenia tend to have poorer premorbid functioning than their female counterparts. Other authors^19^ have also posited that males experience more decline in their premorbid functioning over the epoch ages. The associative and even predictive association found between both in this work lends credence to the current established findings. This may be explained by the fact that females generally exhibit better premorbid social functioning,^19^ have higher frequency of marital stability premorbidly^19^ and have later age of illness onset.^13^ Lenroot and Giedd^48^ as well as Tuszynski and others^49^ have also suggested that females’ earlier maturity – both cognitively and emotionally –^48,49^ might have protected them from the insult to the brain at the same period in which their male counterparts were vulnerable. Even if the insult occurred later, they might have matured beyond the age in which such insult would have a deleterious effect on their premorbid functioning.

Findings from the multiple linear regression analysis in this study demonstrated that the later epoch ages (late adolescence and adulthood) consistently demonstrated a significant relationship with the positive, negative and generalised subscales. Consistent with this picture, Larsen et al.^50^ in a study conducted amongst 40 people reported that the age epochs close to the onset of the illness had worse premorbid adjustment scores than the earlier developmental periods, that is, the level of premorbid adjustment seemed to deteriorate as the patient advanced in age. They proposed that although the insult could have occurred during the earliest period of a patient’s life, illness development occurs around the mid-adolescent period but fully takes off around the late adolescent/adulthood period with the resultant clinical expression almost immediately. This explanation seems plausible, given the results obtained in our study.

Studies have shown that poor premorbid adjustment is associated with severity of symptoms^51,52^ and may even be a predictor of the latter.^51^ It was, therefore, not surprising when this study revealed the association between good premorbid adjustment and no or bordeline illness severity. This means that patients who attained good socio-sexual functioning before the onset of the illness seemed not to be so ill or unable to function in general spheres of life as at the time of this survey. As a corollary, the association between poor premorbid adjustment and poor current functioning has been documented.^53^

Overall, this work has highlighted the premorbid adjustment of a group of outpatients with schizophrenia. However, given the modest amounts of explanatory power attributable to premorbid functioning at different epoch ages in this study, it may be reasonable to suggest that other unmeasured factors play active roles in the evolution of schizophrenia. Thus, premorbid functioning need not be seen as the singular predictor of illness or illness outcome in schizophrenia. Despite the strengths of this work, other potential variables related to premorbid functioning such as investigation for biomarkers or neurological abnormalities were not conducted. Also, the possible history of febrile convulsions, seizure disorder, head trauma or psychoactive substance use prior to the onset of the illness was not obtained. These may also have been important contributors to the poor premorbid functioning amongst the sample.^53,54,55,56^

As noted earlier, a large proportion of this study population appeared to be stable both clinically and functionally. Beyond premorbid functioning, additional current factors such as adequate compliance with treatment and good family support may have contributed to this. It may, therefore, be important to investigate the role of some of these factors in illness severity in the future studies.

Cross-sectional studies do not provide a firm basis for determining the direction of causality of events. However, our focus on an earlier period helped to create a temporal ordering of premorbid adjustment and illness-related variables. Notwithstanding this, the possibility of recall bias cannot be ruled out in relation to premorbid functioning.

Furthermore, the results of the linear regression modelling of the relationship between illness severity and premorbid functioning must be interpreted with caution because of non-normality and non-linearity observed in both the illness severity measures and the PAS scores. Attempts at log transformation did not yield any normalisation of the data. Thus, whilst our analysis maintained fidelity to other assumptions of linear regression, the aforementioned assumptions were not followed.

It is also important to note that this work was conducted in a southwest Nigerian hospital with members of one ethnic group being over-represented. Thus, its generalisation to other parts of the country must be made with caution.

## Conclusion

Poor premorbid adjustment was considerably present in this sample of patients with schizophrenia, and it was associated with male gender. In other words, clinicians should bear in mind that male patients with the illness may have likely had poorer premorbid functioning than females, especially in terms of marital and occupational achievement. These factors should also be considered when formulating rehabilitation goals as males may have lower room for improvement (and hence remission). Antenatal care providers should also be interested in exploring the other factors that may be responsible for insults to developing brains as well as their pathogenesis in the development of schizophrenia. Therefore, antenatal studies that encompass follow-up into adulthood period should be one of the focus of future researchers.
